# Understanding Genetic Diversity of Sorghum Using Quantitative Traits

**DOI:** 10.1155/2016/3075023

**Published:** 2016-06-13

**Authors:** Sweta Sinha, N. Kumaravadivel

**Affiliations:** Department of Plant Molecular Biology and Biotechnology, Centre for Plant Molecular Biology, Tamil Nadu Agricultural University, Coimbatore, Tamil Nadu 641003, India

## Abstract

Sorghum is the important cereal crop around the world and hence understanding and utilizing the genetic variation in sorghum accessions are essential for improving the crop. A good understanding of genetic variability among the accessions will enable precision breeding. So profiling the genetic diversity of sorghum is imminent. In the present investigation, forty sorghum accessions consisting of sweet sorghum, grain sorghum, forage sorghum, mutant lines, maintainer lines, and restorer lines were screened for genetic diversity using quantitative traits. Observations were recorded on 14 quantitative traits, out of which 9 diverse traits contributing to maximum variability were selected for genetic diversity analysis. The principle component analysis revealed that the panicle width, stem girth, and leaf breadth contributed maximum towards divergence. By using hierarchical cluster analysis, the 40 accessions were grouped under 6 clusters. Cluster I contained maximum number of accessions and cluster VI contained the minimum. The maximum intercluster distance was observed between cluster VI and cluster IV. Cluster III had the highest mean value for hundred-seed weight and yield. Hence the selection of parents must be based on the wider intercluster distance and superior mean performance for yield and yield components. Thus in the present investigation quantitative data were able to reveal the existence of a wide genetic diversity among the sorghum accessions used providing scope for further genetic improvement.

## 1. Introduction

Sorghum (*Sorghum bicolor*) is the world's fifth most important cereal, after wheat, rice, maize, and barley [1, 2]. It is a major food crop in Sub-Saharan Africa and South Asia and is the staple food for the most food insecure people in the world [3]. Besides being an important food, feed, and forage crop, it provides raw material for the production of starch, fiber, dextrose syrup, biofuels, alcohol, and other products. Sorghum was domesticated in African continent, particularly in Ethiopia, from where it was introduced to other regions of the world with diverse agroclimatic conditions [4]. Therefore a wide diversity is found within and among the sorghum cultivars at both phenotypic and genotypic level [5, 6]. Knowledge of genetic diversity of a crop usually helps the breeder in choosing desirable parents for the breeding program and gene introgression from distantly related germplasm. The more diverse genotypes or accessions can be crossed to produce superior hybrids with resistance to abiotic and biotic stresses. Understanding the wealth of genetic diversity in sorghum will facilitate further improvement of this crop for its genetic architecture [7].

Genetic diversity in the crop species is the gift of nature and arises due to geographical separation or due to genetic barriers to crossability. Morphological traits are conventional tools to analyze the genetic diversity. Morphological assays generally require neither sophisticated equipment nor preparatory procedures. They are generally simple and inexpensive to score. These easily observable quantitative morphological traits are useful tool for preliminary evaluation, because they offer a fast and useful approach for assessing the extent of diversity. Over the years, a number of studies have dealt with estimating genetic diversity in cultivated sorghum using morphological traits [8–14]. The use of morphological traits is the most common approach utilized to estimate relationships between genotypes. The genetic variability of cultivated species/varieties and their wild relatives together forms a potential and continued source for breeding new and improved crop varieties. A better understanding of genetic diversity in sorghum will facilitate crop improvement. Therefore there is a need to evaluate the available accessions for genetic diversity. In the present study, an attempt has been made to determine the extent of diversity among forty sorghum accessions using the quantitative traits.

## 2. Materials and Methods

### 2.1. Plant Material

The plant materials consisted of forty accessions of sorghum collected from different parts of Tamil Nadu (Table 1). Among these forty accessions, four of accessions were sweet sorghum, seventeen were grain sorghum, two were forage sorghum, ten were mutant populations, three were B-lines, and the remaining four accessions were R-lines.

### 2.2. Methods

The forty sorghum accessions were raised in a randomized block design (RBD) with two replications for one season at Millet Breeding Station, TNAU, Coimbatore, Tamil Nadu. Each accession was raised in a single row of 5 meters length by adopting a spacing of 45 cm × 15 cm. All the recommended agronomic packages of practices such as irrigation, fertilizer doses, and crop protection management were adopted during the entire crop period. In each replication, five random plants were chosen and the observations were recorded on fourteen quantitative traits at the time of maturity except days to 50 percent flowering. Observations consisted of days to 50% flowering (DFL), days to maturity (DMY), plant height (PHT), panicle length (PNL), panicle width (PWD), leaf length (LFL), leaf breadth (LFW), number of leaves per plant (NPL), stem girth (SGT), number of primary branches per panicle (NPB), hundred-seed weight (HWT), yield per plant (YLD), panicle weight (PWT), and dry matter production (DMP). The mean values were utilized for statistical analysis to assess the genetic diversity among the accessions.

### 2.3. Statistical Analysis of Quantitative Traits

Prior to analysis the data were standardized to zero mean and unit variance, because various traits were measured on very different scales. The descriptive statistics, analysis of variance, and correlation coefficients were computed for all the fourteen quantitative traits using Microsoft Excel 2003. Factor analysis was performed to know which trait is contributing maximum variability. Principal component analysis of the traits was employed to examine the percentage contribution of each trait to total genetic variation. Agglomerative hierarchical clustering was performed on the Euclidean distance matrix utilizing Ward's linkage method. These analyses were done using MINITAB software version 13.

## 3. Result and Discussion

The analysis of variance using a randomized block design indicated significant variation for all 14 quantitative traits (result not shown) investigated indicating that there was a high level of genetic diversity among the sorghum accessions.

### 3.1. Descriptive Statistics

Statistical analysis was carried out with the data on fourteen quantitative traits to assess the variability pattern (Table 2). Among all the traits investigated, dry matter production recorded maximum value of mean, standard error, variance, standard deviation, coefficient of variation, and range. The descriptive statistics of fourteen quantitative indicated the existence of morphological diversity among the sorghum accessions, providing scope for improvement through hybridization and selection. The coefficient of variation for yield, panicle weight, and dry matter production were high denoting susceptibility to environmental fluctuation influencing their expression to some degree.

### 3.2. Correlation Analysis

The correlation coefficients of fourteen quantitative traits were used in characterizing the forty sorghum accessions. The correlation coefficients of fourteen quantitative traits estimated are presented in Table 3. The high positive and significant correlation value was obtained for panicle weight and hundred-seed weight with yield [15, 16]. The yield was also positively and significantly associated with leaf length, leaf breadth, and number of leaves. Leaf length, leaf breadth, and number of leaves could contribute to quantity of food synthesized by the plant during photosynthesis and have direct effects on yield. From these results it is evident that these traits are associated with grain yield and are intercorrelated among them. Thus, the selection in any one of these yield attributing traits will lead to increase in the other traits, thereby finally enhancing the grain yield. Hence, selection for traits like leaf length, leaf breadth, number of leaves per plant, panicle weight, and hundred-seed weight may also be given importance along with yield.

### 3.3. Factor Analysis

**Figure 1:**
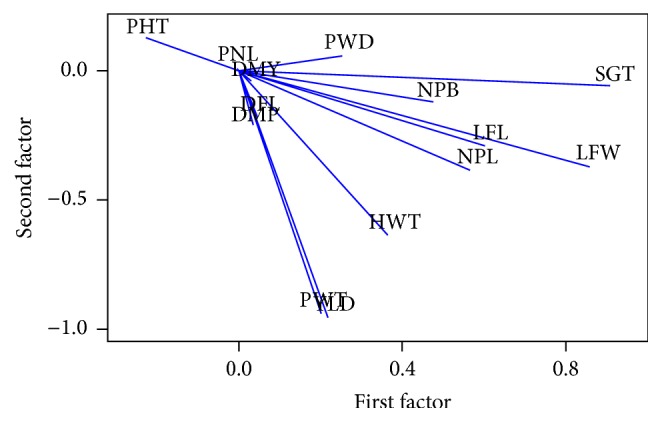
Loading plot of quantitative traits based on factor analysis.

Factor analysis was performed in order to reduce a large set of phenotypic traits to a more meaningful smaller set of traits and to know which trait is contributing to maximum variability because genetic improvement depends on the magnitude of genetic variation. Factor analysis provides an exact picture of variability contributed to by each trait. Thus, on the basis of factor analysis, the quantitative traits that are contributing maximum variability to the first three factors are selected for principal component analysis (Table 4). The first three factors are contributing to 57% of the total variance observed. The first factor had high contributing factor loading from stem girth, leaf breadth, leaf length, number of leaves per plant, and number of primary branches per panicle and contributed to 20.1% of the total variation. The second factor had high contributing loading from yield, panicle weight, and hundred-seed weight and contributed to 19.2% of the total variation. The third factor had high contributing loading from panicle length, panicle width, plant height, hundred-seed weight, and leaf length and contributed to 17.7% of total variation. Distribution of biometrical traits in first two factors is shown in loading plot (Figure 1). The loading plot clearly showed that the traits days to maturity, panicle length, days to 50 percent flowering, panicle weight, and dry matter production had contributed low variability towards genetic variation.

### 3.4. Principal Component Analysis

**Figure 2:**
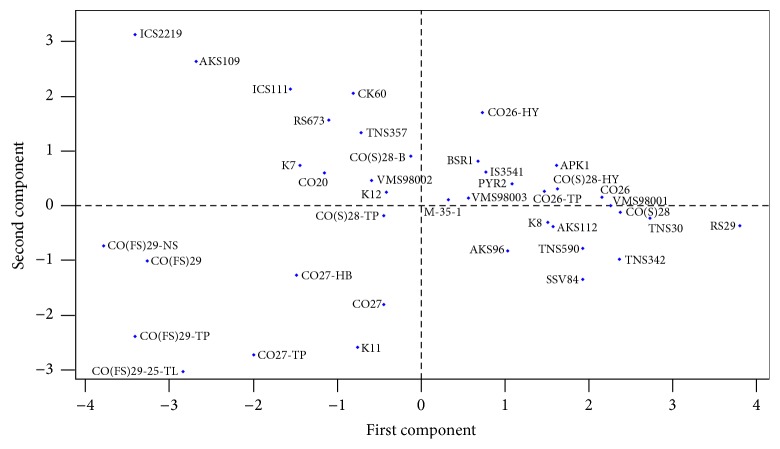
Distribution of sorghum accessions for first two principal components based on nine quantitative traits.

A set of nine diverse quantitative traits were selected from the fourteen traits, namely, stem girth, leaf breadth, leaf length, number of leaves per plant, number of primary branches per panicle, yield, hundred-seed weight, panicle width, and plant height, and were used to group the accessions based on principal component. The first three principal components accounted for 73.2% of the total variance (Table 5). The first principal component (PC1) accounted for 41.7% of total variance and had high contributing factor loading from leaf breadth, stem girth, number of leaves per plant, hundred-seed weight, and yield. The second principal component (PC2) had high contributing factor loading from panicle width, plant height, leaf length, and hundred-seed weight and contributed to 21.8% of the total variation. The third principal component (PC3) accounted for 9.7% of the total variation, with high factor loading for number of primary branches per panicle, stem girth, plant height, yield, and leaf length. The PCA analysis revealed that the panicle width, stem girth, and leaf breadth contributed maximum towards divergence. The score plot of 40 accessions based on the first two principal components is presented in Figure 2. Accessions from similar geographical locations were distributed in different groups. This was exhibited by the accessions of Virinjipuram, Aruppukottai, and Coimbatore. The distribution pattern also indicated the existence of significant amount of variability among the grain sorghum.

### 3.5. Cluster Analysis

**Figure 3:**
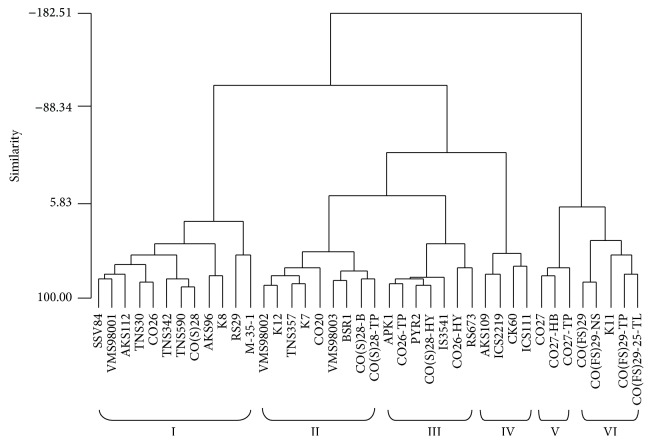
Dendrogram of sorghum accessions based on nine quantitative traits.

Agglomerative hierarchical clustering performed on the Euclidean distance matrix utilizing Ward's linkage method and resulting dendrogram is presented in Figure 3. The forty sorghum accessions formed six clusters at 25.04% similarity level. Among the different clusters, the cluster size varied from 3 to 12. The maximum number of accessions was included in cluster I having 12 accessions and the minimum number in cluster VI having 3 accessions. Cluster I consisted of sweet sorghum, grain sorghum, and restorer lines. Cluster II consisted of sweet sorghum, grain sorghum, and CO(S)28 mutants. Cluster III consisted of grain sorghum, restorer lines, and CO26 mutant and CO(S)28 mutant. Cluster IV consisted of grain sorghum and maintainer lines. Cluster V and cluster VI consisted of forage sorghum and its mutant. The clustering pattern indicated the existence of significant amount of variability among the grain sorghum. The existence of morphological variation was found among sorghum accessions collected from eastern parts of Ethiopia using 10 morphological traits and variation among the sorghum germplasm [17]. The morphological diversity was observed among sorghum accessions as well as a high level of diversity within each region and was distributed with geographical origin using Sudanese sorghum landraces [18]. Also a high level of morphological and genetic variability was found in sorghum varieties from Burkina Faso [19].

The highest intercluster distance was observed between cluster IV and VI (5.148); the accessions from those clusters if chosen for hybridization program may give broad spectrum of variability in segregating generation (Table 6). The lowest intercluster distance was observed between II and III (2.133). The clusters contributing maximum to the divergence were given greater emphasis for deciding the type of cluster for the purpose of further selection and the choice of the parents of hybridization [20].

The cluster mean of the six similarity cluster groups in the 40 sorghum accessions are presented in Table 7. Cluster I had the highest mean values for leaf length (71.17), leaf breadth (8.41), number of leaves per plant (9.42), and stem girth (5.84). Cluster II showed moderate mean values for leaf length, leaf width, stem girth, and hundred-seed weight. Cluster III had the highest mean values for hundred-seed weight (2.99) and yield (50.00). Cluster IV had the lowest mean values for plant height (97.03) and panicle width (4.00). Cluster V had the highest mean values for panicle width (9.26). Cluster VI had the highest mean values for plant height (255.92) and number of primary branches per panicle (59.83). Based on the cluster means, the important cluster is cluster III which had the highest mean values for hundred-seed weight and yield. Hence the accessions falling under these clusters could be used as the parents for hybridization program.

## 4. Conclusion

This study supports that quantitative traits are useful tool for preliminary evaluation of genetic diversity. Correlation studies clearly showed that the traits, namely, leaf length, leaf breadth, number of leaves per plant, panicle weight, and hundred-seed weight, had significant and positive association with yield. The principle component analysis and hierarchical cluster analysis grouped the sorghum accessions under six clusters. Hence, selection of parents must be based on the wider intercluster distance and superior mean performance for yield and yield components. Based on the quantitative trait data, the accessions, namely, CO20 and Paiyur2, were found to be superior for earliness and APK1 and CO26-high yield mutant for grain yield. Therefore these accessions should be utilized in further breeding program for developing superior varieties.

## Figures and Tables

**Table 1:** List of sorghum accessions.

S number	Accessions	Pedigree	Source
*Sweet sorghum*			
1	SSV84	Selection from zera-zera sorghum IS2356	Agricultural Research Station, Virinjipuram
2	VMS98001	Selection from CSV15-1	Agricultural Research Station, Virinjipuram
3	VMS98002	Selection from NSS04-1	Agricultural Research Station, Virinjipuram
4	VMS98003	Selection from 208-1	Agricultural Research Station, Virinjipuram

*Grain sorghum*			
5	APK1	Hybrid derivative of TNS30 × CO26	Regional Research Station, Aruppukottai
6	BSR1	Multiple cross derivative (SC108-3 × ICSV4) 16-3-1 × (MR-801 × R2751) 4-1-1	Agricultural Research Station, Bhavanisagar
7	Paiyur2 (PYR2)	Pureline selection from IS15845	Regional Research Station, Paiyur
8	AKS96	Culture from Aruppukottai	Regional Research Station, Aruppukottai
9	AKS109	Culture from Aruppukottai	Regional Research Station, Aruppukottai
10	AKS112	Culture from Aruppukottai	Regional Research Station, Aruppukottai
11	TNS30	(CO18 × CO22) × 1022	Millets Breeding Station, Coimbatore
12	TNS342	Culture from Coimbatore	Millets Breeding Station, Coimbatore
13	TNS357	SPV1010 × SPV881	Millets Breeding Station, Coimbatore
14	TNS590	Culture from Coimbatore	Millets Breeding Station, Coimbatore
15	K7	K3 × M35-1	Millets Breeding Station, Coimbatore
16	K8	IS12611 × SV108	Millets Breeding Station, Coimbatore
17	K11	K7 × A6552	Millets Breeding Station, Coimbatore
18	K12	Culture from Coimbatore	Millets Breeding Station, Coimbatore
19	CO(S)28	CO25 × SPV942	Millets Breeding Station, Coimbatore
20	CO20	CO1 × Bonganhilo	Millets Breeding Station, Coimbatore
21	CO26	Derivative of MS8271 × IS3691	Millets Breeding Station, Coimbatore

*Forage sorghum*			
22	CO27	CO11 × *S. halepense*	Department of Forage Crops, Coimbatore
23	CO(FS)29	TNS30 × *S. sudanense*	Department of Forage Crops, Coimbatore

*CO26 mutants*			
24	CO26-tall plant mutant (TP)	*γ* ray (40 kR) induced mutant of CO26	Millets Breeding Station, Coimbatore
25	CO26-high yield mutant (HY)	*γ* ray (40 kR) induced mutant of CO26	Millets Breeding Station, Coimbatore

*CO(S)28 mutants*			
26	Co(S)28-bold grain mutant (B)	*γ* ray (35 kR) induced mutant of CO(S)28	Millets Breeding Station, Coimbatore
27	CO(S)28-high yield mutant (HY)	*γ* ray (35 kR) induced mutant of CO(S)28	Millets Breeding Station, Coimbatore
28	CO(S)28-tall plant mutant (TP)	*γ* ray (35 kR) induced mutant of CO(S)28	Millets Breeding Station, Coimbatore

*CO27 mutants*			
29	CO27-tall plant mutant (TP)	*γ* ray (50 kR) induced mutant of CO27	Millets Breeding Station, Coimbatore
30	CO27-high biomass mutant (HB)	*γ* ray (50 kR) induced mutant of CO27	Millets Breeding Station, Coimbatore

*COFS29 mutants*			
31	CO(FS)29-nonshattering mutant (NS)	*γ* ray (60 kR) induced mutant of CO(FS)29	Millets Breeding Station, Coimbatore
32	CO(FS)29-tall plant mutant (TP)	*γ* ray (60 kR) induced mutant of CO(FS)29	Millets Breeding Station, Coimbatore
33	CO(FS)29-25 high tiller mutant (TL)	*γ* ray (60 kR) induced mutant of CO(FS)29	Millets Breeding Station, Coimbatore

*Maintainer lines*			
34	CK60	Culture from Coimbatore	Millets Breeding Station, Coimbatore
35	ICS111	Culture from Coimbatore	Millets Breeding Station, Coimbatore
36	ICS2219	Culture from Coimbatore	Millets Breeding Station, Coimbatore

*Restorer lines*			
37	IS3541	Culture from Coimbatore	Millets Breeding Station, Coimbatore
38	RS29	Culture from Coimbatore	Millets Breeding Station, Coimbatore
39	RS673	Culture from Coimbatore	Millets Breeding Station, Coimbatore
40	M-35-1	Culture from Coimbatore	Millets Breeding Station, Coimbatore

**Table 2:** Descriptive statistics of quantitative traits.

	DFL	DMY	PHT	PNL	PWD	LFL	LFW	NPL	SGT	NPB	HWT	YLD	PWT	DMP
Mean	66.20	104.66	182.99	24.91	7.07	62.26	6.76	8.72	4.69	47.29	2.30	35.66	47.97	450.36
Standard error	0.88	0.83	7.50	1.10	0.33	1.85	0.26	0.13	0.16	1.62	0.14	1.83	2.18	27.75
Standard deviation	5.59	5.26	47.44	6.95	2.08	11.68	1.66	0.80	1.03	10.23	0.86	11.58	13.76	175.49
Sample variance	31.22	27.67	2250.37	48.27	4.32	136.50	2.77	0.65	1.06	104.57	0.74	134.14	189.31	30797.12
Range	26.00	24.00	209.60	30.90	9.95	55.05	6.55	4.05	3.80	47.50	3.45	41.50	52.00	673.50
Coefficient of variation	8.44	5.03	25.92	27.89	29.43	18.77	24.62	9.22	21.96	21.62	37.37	32.47	28.68	38.97

**Table 3:** Pearson's correlation coefficients of quantitative traits.

	DFL	DMY	PHT	PNL	PWD	LFL	LFW	NPL	SGT	NPB	HWT	YLD	PWT
DMY	0.752^*∗∗*^												
PHT	−0.011	−0.241											
PNL	0.058	0.021	0.580^*∗∗*^										
PWD	−0.054	−0.186	0.564^*∗∗*^	0.596^*∗∗*^									
LFL	0.069	−0.184	0.159	0.253	0.386^*∗*^								
LFW	0.126	0.035	−0.265	−0.076	0.137	0.578^*∗∗*^							
NPL	0.278	0.263	−0.072	0.008	0.052	0.252	0.564^*∗∗*^						
SGT	0.060	0.104	−0.180	0.051	0.264	0.477^*∗∗*^	0.741^*∗∗*^	0.576^*∗∗*^					
NPB	−0.048	−0.299	0.156	−0.171	0.120	0.282	0.525^*∗∗*^	0.309	0.319^*∗*^				
HWT	0.221	0.227	−0.440^*∗∗*^	−0.408^*∗∗*^	−0.189	0.121	0.649^*∗∗*^	0.455^*∗∗*^	0.352^*∗*^	0.343^*∗*^			
YLD	0.230	0.121	−0.147	0.013	0.021	0.361^*∗*^	0.525^*∗∗*^	0.547^*∗∗*^	0.292	0.235	0.668^*∗∗*^		
PWT	0.305	0.138	−0.131	−0.070	−0.018	0.320^*∗*^	0.521^*∗∗*^	0.537^*∗∗*^	0.286	0.286	0.707^*∗∗*^	0.961^*∗∗*^	
DMP	0.153	0.002	0.327^*∗*^	−0.064	0.212	0.078	0.187	0.254	0.176	0.271	0.320^*∗*^	0.264	0.325^*∗*^

^*∗*^ *P* < 0.05, ^*∗∗*^ *P* < 0.01.

**Table 4:** Sorted rotated factor loadings of quantitative traits.

Variable	Factor 1	Factor 2	Factor 3
Stem girth	0.908	−0.057	−0.048
Leaf breadth	0.858	−0.371	0.089
Leaf length	0.602	−0.291	−0.431
Number of leaves per plant	0.565	−0.384	0.005
Yield	0.217	−0.955	−0.019
Panicle weight	0.202	−0.939	0.038
Hundred-seed weight	0.364	−0.636	0.459
Panicle length	−0.009	0.002	−0.885
Panicle width	0.253	0.058	−0.796
Plant height	−0.227	0.129	−0.786
Days to maturity	0.029	−0.04	0.128
Days to 50% flowering	0.045	−0.178	−0.052
Dry matter production	0.035	−0.209	−0.11
Number of primary branches per panicle	0.476	−0.122	0.071
*Variance*	2.81	2.685	2.479
*% variance*	20.1	19.2	17.7
*Cumulative % variance*	20.1	39.3	57.0

**Table 5:** Principal components analysis showing the contribution of 9 characters among the sorghum accessions.

Traits	PC1	PC2	PC3
Stem girth	0.396	−0.113	0.486
Leaf breadth	0.477	−0.004	0.146
Leaf length	0.302	−0.353	0.281
Number of leaves per plant	0.381	0.024	−0.083
Number of primary branches per panicle	0.286	−0.158	−0.055
Yield per plant	0.371	0.117	−0.317
Hundred-seed weight	0.377	0.336	−0.256
Panicle width	0.078	−0.602	0.097
Plant height	−0.112	−0.59	−0.426
*Eigenvalue*	3.753	1.96	0.876
*% variance*	41.7	21.8	9.7
*Cumulative % variance*	41.7	63.5	73.2

**Table 6:** Intercluster distances among sorghum accessions.

	Cluster I	Cluster II	Cluster III	Cluster IV	Cluster V	Cluster VI
Cluster I	0.000					
Cluster II	2.813	0.000				
Cluster III	2.343	2.133	0.000			
Cluster IV	5.088	2.955	4.168	0.000		
Cluster V	5.125	3.823	4.827	4.705	0.000	
Cluster VI	4.260	3.105	4.150	5.148	3.188	0.000

**Table 7:** Characteristic means of six similarity cluster groups of sorghum accessions.

Traits	I	II	III	IV	V	VI
Plant height	178.40	164.68	181.16	97.03	254.45	255.92
Panicle width	7.98	6.61	5.78	4.00	9.26	7.33
Leaf length	71.17	57.54	62.76	46.13	56.74	69.40
Leaf breadth	8.41	6.76	6.74	5.36	3.54	6.50
Number of leaves per plant	9.42	8.28	9.14	8.13	8.15	7.82
Stem girth	5.84	4.37	4.31	4.14	3.36	4.05
Number of primary branches per panicle	50.50	49.00	50.79	32.00	34.81	59.83
Hundred-seed weight	2.68	2.59	2.99	1.94	0.60	1.17
Yield per plant	42.39	30.63	50.00	25.79	26.13	21.67
